# Gene Expression and DNA-Methylation of Bovine Pretransfer Endometrium Depending on Its Receptivity after *In Vitro*-Produced Embryo Transfer

**DOI:** 10.1371/journal.pone.0042402

**Published:** 2012-08-27

**Authors:** Siriluck Ponsuksili, Eduard Murani, Manfred Schwerin, Karl Schellander, Dawit Tesfaye, Klaus Wimmers

**Affiliations:** 1 Research Group ‘Functional Genome Analysis’, Leibniz Institute for Farm Animal Biology, Dummerstorf, Germany; 2 Research Unit ‘Molecular Biology’, Leibniz Institute for Farm Animal Biology, Dummerstorf, Germany; 3 Institute of Animal Science, Animal Breeding and Husbandry Group, University of Bonn, Bonn, Germany; Huazhong Agricultural University, China

## Abstract

Embryonic implantation to establish a pregnancy is a complex process that requires appropriate communication between the embryo and the maternal endometrium. Inadequate uterine receptivity may contribute to the majority of implantation failures. To provide a comprehensive inventory of genes and functional networks that represent the maternal input of the embryo-maternal cross-talk, a longitudinal, holistic study of the endometrial transcriptome in relation to the days of estrous and to the receptivity of the endometrium was performed in bovine. At day 3 of estrous, genes related to cell communication and mitochondrial energy metabolism were differentially expressed among high- and low-receptive endometria (HR, LR); at day 7, transcripts functioning in immune and inflammatory pathways, oxidative stress, and angiogenesis had different abundances. Additionally, temporal transcriptional changes between days 3 and 7 differed considerably among HR and LR. Further, several transcription factors were predicted as relevant for receptivity because they were either differentially expressed among HR and LR animals or are known to be associated with genes we detected to have differential expression. Finally, global DNA methylation varied according to the interaction of receptivity group and day of estrous, and a divergent trend, which correlated with abundance of *DNMT1* transcript, was observed in LR and HR along the estrous cycle days. The study revealed that, even in early estrous, transcripts related to cell communication and response to exogenous stimuli, vascularization, and energy supply show divergent expression and longitudinal temporal regulation in HR and LR. Key components of these molecular pathways are known to be dependent on ovarian hormones that promote uterine receptivity.

## Introduction

Successfully establishing a pregnancy requires a dialogue between the endometrium and the implanting embryo [1], [2]. The endometrium exhibits characteristic morphological and functional changes throughout the estrous cycle, some of which allow the endometrium to receive an embryo. These changes are mainly driven by estrogen and progesterone, but they require fine control of many different genes. Indeed, estrous cycle-specific endometrial expression has been reported in human [3], mouse [4], and bovine [5], [6]. Dynamic changes in gene expression occurring during maternal recognition and implantation are associated with pregnancy success. Aberrant endometrial gene expression during critical periods of pregnancy establishment could result in implantation failure and infertility [7]. Analysis of the endometrium transcriptome could provide a blueprint of expression changes, complementing existing knowledge of temporal changes in the endometrial transcriptome and facilitating a better understanding of conceptus-maternal cross-talk during the peri-implantation period. Similarly, identifying genes that affect endometrial receptivity may uncover important biological processes and the underlying genes and functional networks influencing pregnancy success.

Our previous study revealed that, at day 7 of the estrous cycle, the endometrial gene expression pattern of heifers whose pregnancy resulted in calf delivery was significantly different from that of heifers with pregnancy failure. However, at day 14 of the estrous cycle only subtle differences were observed in their transcriptomes by endometrial receptivity [8]. To refine these results, and to obtain information about expression earlier in estrous, we performed endometrial expression profiling at days 3 (pre-implantation) and 7 of estrous in cows from high and low receptivity groups. Additionally, transcription factor analyses were conducted to identify the cascade of upstream transcriptional regulators inducing gene expression changes in the endometria. Finally, as epigenetic modifications may effect functional changes in the endometrium, we investigated expression of *DNMT1*, *DNMT3A*, and *DNMT3B*—genes coding for the DNA methyltransferases that catalyze methylation—and global methylation status in the bovine endometrium during the estrous cycle. Our study provides a survey of genes, biofunctions, and molecular pathways expressed in the bovine endometrium at early stages of the estrous cycle that are associated with uterine receptivity and implantation success.

## Results

We performed a longitudinal, holistic study of the endometrial transcriptome in relation to both the day of estrous and the receptivity of the endometrium to provide a comprehensive inventory of genes and functional networks representing the maternal contribution that may predict success of pregnancy in bovine. Endometrial biopsies were obtained at days 3 and 7 of the estrous cycle and were grouped retrospectively according to their receptivity, a characteristic determined by pregnancy outcome in the next cycle. RNA pools were hybridized to Affymetrix GeneChip Bovine Genome Arrays containing 24,013 probe sets, of which 9,269 were subjected to further analysis after filtering for consistent ‘present calls’ and variability of expression.

Transcript abundance was compared between high and low receptivity groups (LR and HR) on day 3 or on day 7 of estrous. We also analyzed differences among receptivity groups for changes of expression patterns between days 3 and 7 of the estrous cycle one cycle prior to embryo transfer. Mixed-model analysis of expression levels of the time point effect in the model revealed 5,640 probe sets indicating significant differences of the abundance of transcripts (p<0.05, corresponding to FDR<0.16).

### Comparisons between endometrial receptivity groups on day 3 of estrous cycle

**Figure 1:**
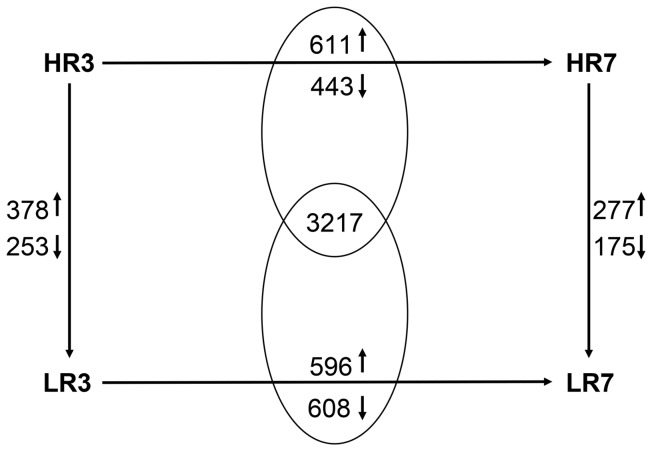
Number of regulated probe-sets in the endometrial biopsies. The numbers at the horizontal arrows indicate the quantity of probe-sets significantly regulated between the adjacent stages within either the HR or the LR group, whereas the numbers in the intersections indicate the quantity of probe-sets commonly regulated between day 3 to day 7 (↑ = higher transcript abundance at day 3, ↓ = lower transcript abundance at day 3) in both the HR and the LR group. The numbers at vertical arrows are the number of probe-sets differentially expressed between HR and LR at the same day of estrous cycle (↑ = higher transcript abundance in the HR groups, ↓ = lower transcript abundance in the HR groups).

**Figure 2:**
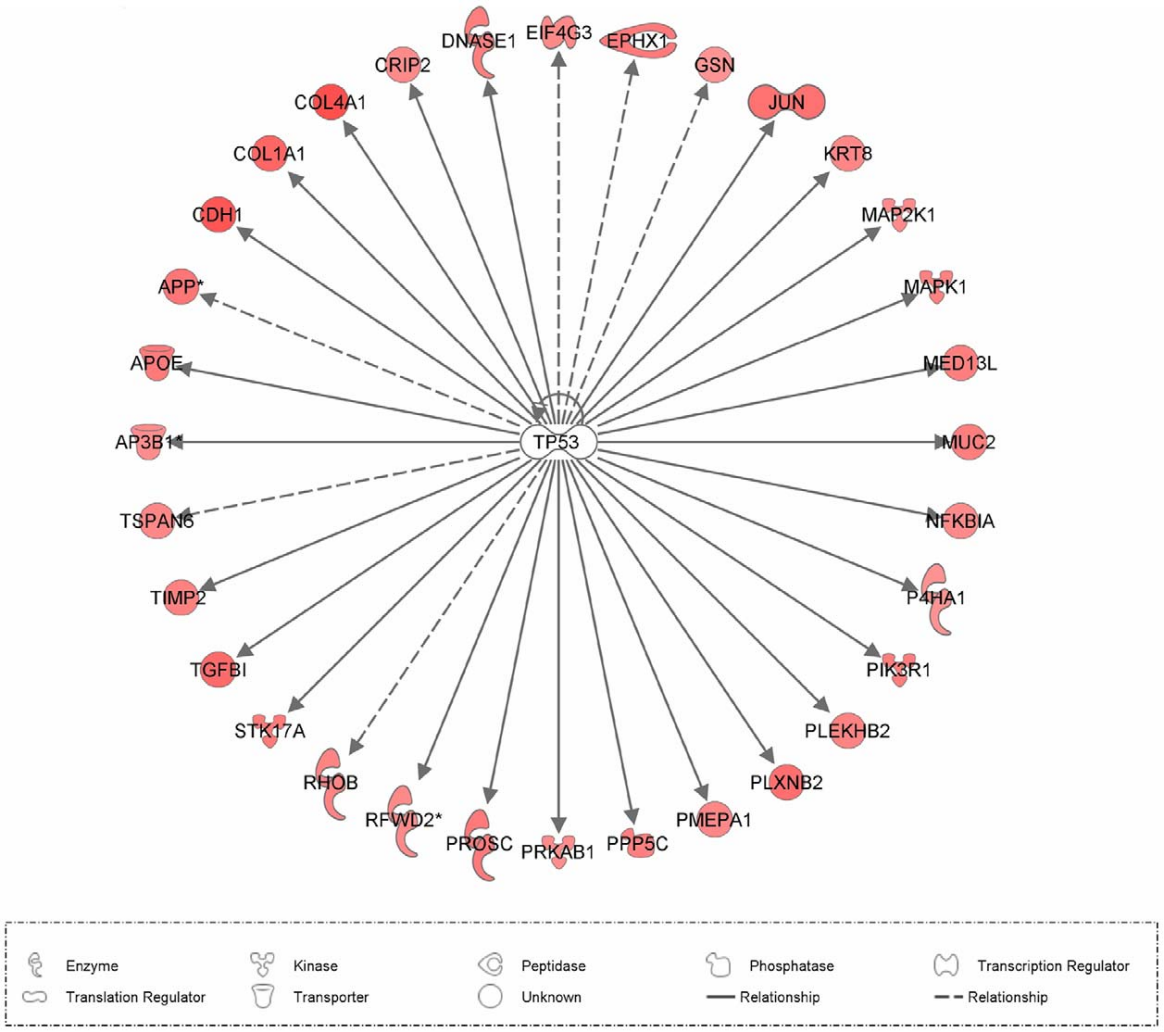
Canonical pathways enriched for transcripts showing different abundance depending on receptivity group and day of estrous cycle. Pathways showing temporal regulation from day 3 to day 7 are given at the horizontal vectors of either the HR (top part) or the LR group (lower part) (↑ = higher transcript abundance at day 3, ↓ = lower transcript abundance at day 3). Pathways of transcript showing different abundance among the Hr and LR at day 3 (left) or day 7 od estrous cycle are displayed at the vertical vectors (↑ = higher transcript abundance in the HR groups, ↓ = lower transcript abundance in the HR groups).

We identified 631 differentially-expressed probe sets (594 genes) between HR and LR endometria at day 3 of estrous (Table S1). Of these, 378 probe sets (358 genes) represented more abundant (HR3>LR3) and 253 probe sets (236 genes) represented less abundant (HR3<LR3) transcripts in the high receptivity group (Figure 1). We assessed the significance of direction of abundance of candidate genes for enrichment in functional annotation groups, as defined in the Ingenuity Pathway Knowledge Base. Genes with higher expression levels in the HR group at day 3 of estrous were often involved in molecular transport, cellular assembly and organization, and cell cycle (Table 1, Figure 2). To further refine the functional annotation of gene sets with higher abundance in the receptive group, their assignment to canonical pathways was explored using the Ingenuity Pathway Knowledge Base. The top three significantly enriched canonical pathways for these transcripts included integrin signaling (*TSPAN3, TSPAN5, CAPNS1, PAK6, MAPK1, RHOB, PIK3R1, ITGAV, PPP1CB, BCAR3, TSPAN6, MAP2K1*), actin cytoskeleton signaling (*IQGAP2, ARHGEF12, PAK6, MAPK1, PIK3R1, PIKFYVE, PPP1CB, GSN, MAP2K1, GNG12, F2, FGF1*) and Rac signaling (*IQGAP2, JUN, PAK6, MAPK1, PIK3R1, PIKFYVE, PARD3, MAP2K1*) (Table 1). Transcripts that were less frequent in the HR group at day 3 of estrous were assigned to cellular assembly and organization, cellular function and maintenance, and energy production (Table 1, Figure 2). The transcripts of these groups were categorized in canonical pathways of mitochondrial dysfunction (*GSR, NDUFA5, COX6A1, NDUFS6, NDUFS2, CYCS, NDUFB1*), ubiquinone biosynthesis (*NDUFA5, NDUFS6, FTSJ1, NDUFS2, NDUFB1*), and oxidative phosphorylation (*NDUFA5, COX6A1, ATP5J2, NDUFS6, NDUFS2, NDUFB1*) (Table 1).

**Table 1:** Molecular and cellular functions of transcripts with different abundance in the HR and LR groups at day 3 of the estrous cycle.

	No. of genes	p-values
**HR3>LR3**		
**Molecular and cellular functions**		
Molecular Transport	65	4.41E-06-1.71E-02
Cellular Assembly and Organization	66	4.97E-06-1.71E-02
Cell Cycle	48	3.98E-05-1.71E-02
**Canonical Pathways**		
Integrin Signaling	12	1.69E-04
Actin Cytoskeleton Signaling	12	3.17E-04
Rac Signaling	8	4.18E-04
**HR3<LR3**		
**Molecular and cellular functions**		
Cellular Assembly and Organization	21	2.93E-05-4.73E-02
Cellular Function and Maintenance	21	2.93E-05-4.73E-02
Energy Production	7	1.22E-03-4.45E-02
**Canonical Pathways**		
Mitochondrial Dysfunction	7	2.35E-04
Ubiquinone Biosynthesis	5	4.25E-04
Oxidative Phosphorylation	6	2.05E-03

### Comparisons between endometrial receptivity groups at time point day 7 of estrous cycle

In total, 452 probe sets (397 genes) were differentially-expressed between high and low receptive endometria at day 7 of the estrous cycle (Table S2). Among these, 277 probe sets (243 genes) were more abundant in the high receptivity group (HR7>LR7) (Figure 1). Most of these genes were categorized in molecular and cellular functions of cellular development, cellular movement, and cellular growth and proliferation (Table 2, Figure 2). The top three canonical pathways were T- and B cell signaling (*TLR2, TLR1, IL18, CD80, TNFSF13, RELB, IL1B*), NF-κB signaling (*TLR2, FGFR3, TLR1, IL18, TGFBR1, RELB, IL1B*) and inositol phosphate metabolism (*PLCD1, INPP1, INPP5F, PIM1, CDK6, GRK5*) (Table 2). The remaining 175 probe sets (154 genes) were less frequent in the high receptivity group at day 7 of estrous (HR7<LR7). The top three biological process categories included cellular assembly and organization, cellular function and maintenance, and cell death (Table 2, Figure 2). Only two canonical pathways were enriched in low receptive endometrium: inhibition of angiogenesis (*GUCY1A3, THBS1, GUCY1B3*) and NRF2-mediated oxidative stress response (*ABCC1, DNAJA4, HSPB8, DNAJC11*) (Table 2).

**Table 2:** Molecular and cellular functions of transcripts with different abundance in the HR and LR groups at day 7 of the estrous cycle.

	No. of genes	p-values
**HR7>LR7**		
**Molecular and cellular functions**		
Cellular Development	78	4.15E-09-5.44E-03
Cellular Movement	57	9.9E-09-5.47E-03
Cellular Growth and Proliferation	86	9.98E-07-5.44E-03
**Canonical Pathways**		
T Cell and B Cell Signaling	7	9.67E-05
NF-êB Signaling	7	5.07E-03
Inositol Phosphate Metabolism	6	7.58E-03
**HR7<LR7**		
**Molecular and cellular functions**		
Cellular Assembly and Organization	18	7.06E-07-4.99E-02
Cellular Function and Maintenance	20	5.08E-06-4.84E-02
Cell Death	29	5.06E-04-4.84E-02
**Canonical Pathways**		
Inhibition of Angiogenesis	3	1.75E-03
NRF2-mediated Oxidative Stress Response	4	3.87E-02

### Temporal regulation from day 3 to day 7 of estrous cycle in the high receptive endometrium

Comparing two cycle stages within the two receptivity groups uncovered significantly temporally-regulated transcripts; the resulting gene lists were then compared between the high and low receptivity groups. The intersection of commonly regulated genes (3,217 probe sets) between those comparisons were discarded because these regulations were likely due to physiological development processes in the estrous cycle that are not associated with divergent receptivity (Figure 1). Between day 3 and day 7 of estrous in the high receptive endometrium, there were 1,054 (611 HR3>HR7; 443 HR3<HR7) probe sets (973 genes) showing varied expression that did not overlap with the low receptive group (Table S3). The top five molecular and cellular functions of 611 transcripts were carbohydrate metabolism, lipid metabolism, small molecule biochemistry, gene expression, and cellular assembly and organization (Table 3, Figure 2). For transcripts more frequent at day 7, the most prominent biological functions were cellular movement, cell death, cell-to-cell signaling and interaction, antigen presentation, and cellular function and maintenance (Table 3, Figure 2). Canonical pathways that were assigned only to day 3 of estrous in the HR group (HR3>HR7) included role of BRCA1 in DNA damage response, ATM signaling, role of CHK proteins in cell cycle checkpoint control, and hereditary breast cancer signaling (Table 3). Genes showing temporal regulation in the high receptive group (HR3<HR7) were assigned to the canonical pathways of TREM1 signaling, graft-versus-host disease signaling, the coagulation system, and glutathione metabolism (Table 3).

**Table 3:** Molecular and cellular functions of transcripts with different changes of abundance between day 3 and day 7 of the estrous cycle in the HR groups.

	No. of genes	p-values
**HR3>HR7**		
**Molecular and cellular functions**		
Carbohydrate Metabolism	6	3.70E-05-2.39E-02
Lipid Metabolism	10	3.70E-05-2.93E-02
Small Molecule Biochemistry	22	3.70E-05-2.39E-02
Gene Expression	90	8.16E-05-2.36E-02
cellular assembly and organization	65	1.31E-04-2.39E-02
**Canonical Pathways**		
Role of BRCA1 in DNA Damage Response	9	6.61E-06
Hereditary Breast Cancer Signaling	11	1.15E-04
ATM Signaling	7	2.26E-04
Role of CHK Proteins in Cell Cycle Checkpoint Control	5	1.058E-03
**HR3<HR7**		
**Molecular and cellular functions**		
cellular movement	68	2.44E-10-4.35E-03
cell-to-cell signaling and interaction	57	5.20E-08-4.35E-03
cell death	93	1.68E-07-4.23E-03
Antigen Presentation	33	2.24E-07-2.89E-03
Cellular Function and Maintenance	30	7.86E-07-4.23E-03
**Canonical Pathways**		
TREM1 Signaling	6	3.19E-04
Graft-versus-Host Disease Signaling	5	1.12E-03
Coagulation system	4	2.96E-03
Glutathione Metabolism	5	3.95E-03

### Temporal regulation from day 3 to day 7 of estrous cycle in the low receptive endometrium

In the low receptive group, 1,204 probe sets (1066 genes) showed temporal regulation (Table S4). Of these, 596 probe sets (521 genes) represented transcripts more abundant at day 3 (LR3>LR7) (608 probe sets (554 genes) at day 7 (LR3<LR7)) (Figure 1). Transcripts that were more frequent in day 3 than day 7 in LR were assigned to biofunctions of gene expression, cellular growth and proliferation, cellular development, cell cycle, and cell-to-cell signaling and interaction (Table 4, Figure 2). A significant number of these genes were associated with prolactin signaling (*SOCS1, STAT5A, PLCG2, CREBBP, STAT3, PIK3R4, NR3C1*), lysine degradation (*PLOD2, KAT2B, WHSC1L1, AASDHPPT, WHSC1, ECH1*), and T-cell receptor signaling (*PTPRC, CD247, LCK, LAT, IKBKE, PPP3CC, PIK3R4*) (Table 4). Genes more highly expressed at day 7 were assigned to biofunctions of gene expression, RNA post-transcriptional modification, cellular growth and proliferation, molecular transport, and protein synthesis (Table 4, Figure 2). A significant number of these genes belonged to canonical pathways of mitochondrial dysfunction (*HSD17B10, SDHA, NDUFV1, COX6A1, ATP5A1, APP, GSR, NDUFS1, TXN2, UQCRC2, CYC1, UQCRC1, AIFM1, PSEN1*), estrogen receptor signaling (*MAPK1, PHB2, GTF2F1, GTF2A1, SHC1, CDK8, POLR2C, NCOA2, MED13L, NCOR1, ESR2, PPARGC1A, POLR2K*) and actin cytoskeleton signaling (*PIK3CA, TIAM1, ARHGEF12, PFN1, MAPK1, PIKFYVE, PPP1CB, APC, FGF1, SHC1, IQGAP2, VCL, ACTG2, PIP4K2A*) (Table 4).

**Table 4:** Molecular and cellular functions of transcripts with different changes of abundance between day 3 and day 7 of the estrous cycle in the LR groups.

	No. of genes	p-values
**LR3>LR7**		
**Molecular and cellular functions**		
Gene Expression	98	2.40E-05-5.48E-03
Cellular Growth and Proliferation	113	3.45E-07-1.61E-02
Cellular Development	98	1.30E-06-1.61E-02
Cell cycle	62	5.19E-06-1.33E-02
Cell-To-Cell Signaling and Interaction	33	5.19E-06-1.61E-02
**Canonical Pathways**		
Prolactin Signaling	7	6.23E-04
Lysine Degradation	6	1.33E-03
T cell receptor signaling	7	3.27E-03
**LR3<LR7**		
**Molecular and cellular functions**		
Gene Expression	111	2.34E-08-2.48E-02
RNA Post-Transcriptional Modification	16	2.68E-06-7.50E-03
Cellular Growth and Proliferation	125	1.01E-05-2.48E-02
Molecular Transport	89	2.64E-05-2.48E-02
Protein Synthesis	47	2.82E-05-2.38E-02
**Canonical Pathways**		
Mitochondrial Dysfunction	14	6.12E-06
Estrogen Receptor Signaling	13	1.63E-05
Actin Cytoskeleton Signaling	14	8.96E-04

### Quantitative real-time RT-PCR validation of microarrays

Quantitative real-time RT-PCR was used to confirm differential expression indicated by microarray expression patterns. Several differentially-expressed genes were selected for validation, and three housekeeping genes were used for normalization (*GAPDH, RPS13, SUZ12*). Most of the genes analyzed by qRT-PCR confirmed the direction of differential regulation obtained by microarray analysis. In 80% of the genes that we validated by qRT-PCR, significant expression differences in mRNA levels between the treatment groups were identified by both qRT-PCR and microarray analysis. Correlations between expression values of microarray and qRT-PCR were highly significant (Table 5). This suggests that our microarray data are reliable, although we used a pool of biopsies for microarray compared to individual biopsies by qRT-PCR.

**Table 5:** Comparison of microarray data and qRT-PCR of selected transcripts.

	qRT-PCR_individual						Array_pool						Correlation	
	LSMEAN of relative expression levels and *p-values*						LSMEAN of log2 expression levels and *p-values*						qRT-PCR_vs_Array	
Gene	HR	LR	*p-value*	d3	d7	*p-value*	HR	LR	*p-value*	d3	d7	*p-value*	R	*p*-value
DDX46	119842.7	116256.6	0.752	133304.9	102794.3	0.011	8.6	8.7	0.394	8.8	8.5	0.049	0.636	0.026
KRT5	23695.9	6018.3	0.148	1530.5	28183.8	0.032	8.4	6.9	0.006	6.1	9.2	<.0001	0.899	<.0001
PTGES	66422.5	53800.4	0.323	70944.1	49278	0.094	7.0	6.9	0.429	7.1	6.8	0.177	0.706	0.010
PTGS2	7303.3	9972.8	0.646	2732.7	14543.4	0.048	7.5	8.0	0.414	6.4	9.1	0.002	0.873	<.0001
AP2S1	18037.3	16310.0	0.052	14799.2	19548.1	<.0001	6.4	6.6	0.010	6.4	7.0	0.001	0.636	0.026
HPGD	6447.0	11289.9	0.059	3134.0	14602.9	<.0001	8.5	8.8	0.256	7.8	9.5	<.0001	0.911	<.0001
ESRRA	7530.6	6428.1	0.078	5118.8	8839.9	<.0001	7.5	7.2	0.026	7.2	7.5	0.006	0.711	0.010
TP53	38298.8	25727.9	<.0001	40947.4	23079.3	<.0001	8.5	8.4	0.417	8.7	8.2	0.002	0.836	<.0001
PIP	1986.9	2801.3	0.093	930.3	3858.0	<.0001	7.5	8.3	0.001	6.8	8.9	<.0001	0.826	<.0001
SLK	22498.6	16804.0	0.020	17943.0	21360	0.153	7.4	6.8	<.0001	6.8	7.3	0.004	0.607	0.036
ATP1B3	46554.1	30485.9	<.0001	36087.1	40953	0.245	6.1	5.5	<.0001	5.6	6.0	0.004	0.842	<.0001

### Transcription factor analysis

**Figure 3:**
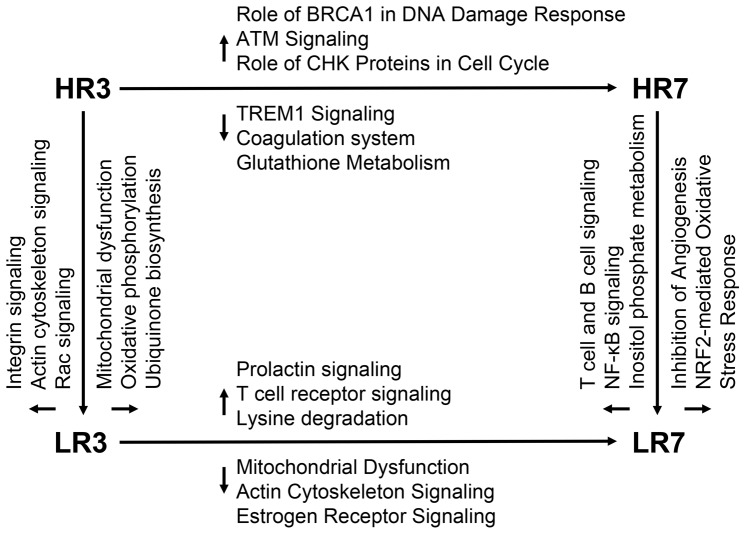
Target genes of TP53 as predicted by IPA.

The study of factors responsible for gene expression changes was done by using transcription factor prediction based on expression data (Table 6). We listed transcription factors that received a predicted activation state due to the direction of regulation of target genes in our data set, consistent with knowledge of their effects on the respective genes in the Ingenuity Pathway Knowledge Base. Additionally, we listed transcription factors that themselves showed differential expression among the experimental groups. At day 3 of estrous, the expression levels of 32 target genes that were more abundant in HR were predicted to be regulated by transcription factor TP53 (overlap p-value 1.46E-04) (Figure 3). Indeed, 19 of 32 genes had expression directions consistent with activation of TP53 (Z-score 2.293). The expression of 14 target genes in the HR group at day 7 of estrous cycle was predicted to be regulated by FOS (overlap p-value 1.39E-03); FOS was predicted to be inhibited (Z-score -2.497). Transcripts showing temporal regulation from day 3 to day 7 of estrous in HR were predicted to be regulated by TP53, HTT, PPARA, and SATB1. Transcription factors TP53 and HTT were predicted to be activated at day 3 in pregnant (HR) individuals, whereas PPARA and SATB1 were predicted to be inhibited at day 3 and day 7 of estrous, respectively. Transcripts in temporal regulation from day 3 to day 7 of estrous in LR were predicted to be regulated by CEBPA, FOS, MYCN, and TP53. Transcription factors CEBPA and TP53 were predicted to be inhibited at day 3 and day 7 of estrous cycle in LR, respectively. Transcription factors FOS and MYCN were predicted to be activated at day 7 of estrous cycle in LR. The change in expression levels of numerous transcript factors and of their downstream target genes was statistically significant (Table 6).

**Table 6:** Transcription factors associated with the differential transcript abundance among the experimental groups.

comparisons	transcription factor	predicted activation state	Z-score^1^	fold change of transcript abundance	p-value of overlap^2^	target molecules
HR3>LR3	TP53	Activated	2.293		1.46E-04	AP3B1,APOE,APP,CDH1,COL1A1,COL4A1,CRIP2,DNASE1,EIF4G3,EPHX1, GSN,JUN,KRT8,MAP2K1,MAPK1,MED13L,MUC2,NFKBIA,P4HA1,PIK3R1, PLEKHB2,PLXNB2,PMEPA1,PPP5C,PRKAB1,PROSC,RFWD2,RHOB, STK17A,TGFBI,TIMP2,TSPAN6
HR7>LR7	FOS	Inhibited	−2.497		1.39E-03	A2M,AQP3,ATF7IP,BCAR3,CADM1,CAST,FN1,KRT13,LAMA3,MGST1, S100A8,S100A9,SELENBP1,TBL1X
	RELB			1.254	1.69E-02	IL1B,NOS2,RELB
	PYCARD			1.642	2.01E-03	IL18,IL1B
	TFAP2A			1.972	3.32E-03	ANXA1,CXCL2,CYP11A1,KRT5,SESN3
HR7>HR3	SATB1	Inhibited	−2.374		9.67E-02	CD69,GATA3,GPT2,SPI1
HR3>HR7	HTT	Activated	2.020		1.45E-02	AQP1,ATP2C1,CDK2AP1,COL16A1,COL18A1,COL4A1,CSNK1E,EBF1,FHL1,GAS7,IGFBP5,JUN,MEIS2,MMP2,PER2,PPRC1,PTPN23,RAB17,SCAMP5, SERPINH1,SESN1,SETDB1,TP53,UCHL1,VIPR2
	PPARA	Inhibited	−2.253		1.13E-01	C3,C8A,CYP2B6,IGFBP5,PCK1
	TP53	Activated	2.033	1.459	1.29E-03	ANTXR1,ASF1B,C1QC,COL18A1,COL4A1,CRIP2,CYR61,DNASE1,F2R,FHL1,GLB1,HSP90AA1,IGFBP4,IGFBP5,IGFBP7,IQCB1,JUN,LATS2,LRRC17, MAP2K4,MFAP2,MMP2,MPDZ,MSH6,PAWR,PCBP4,PIK3R1,PLXNB2, PMEPA1,PRKAB1,RAD50,RHOB,RUVBL2,SESN1,SIVA1,TFPI2,TP53,TP73
	SPI1			−1.445	3.32E-02	CEBPA,CSF2RA,IL18,SPI1,TLR2,TREM1
	IRF5			−1.396	2.20E-02	CCL4,IRF5,RSAD2
	CEBPA			−1.304	8.07E-04	ANXA1,AQP5,BCL2A1,CA2,CEBPA,CSF2RA,DYNLT3,GSTA1,HCAR3,HSPA5,IL8,SFTPD,SPI1,TFAP2A
	CRTC1			−1.203	1.72E-02	NQO1
	PYCARD			−1.136	4.23E-03	IL18,IL8
	TFAP2A			−1.069	1.07E-04	ANXA1,CEBPA,DCBLD2,LIPA,PLA2G16,PLAU,PLAUR,RAB27B
	MYB			1.017	1.33E-02	CD34,IGFBP5,JUN,MYB,VIM
	PHF1			1.215	1.89E-05	HOXA10,HOXA11,HOXA9,HOXD10,HOXD9
	HOXA5			1.317	9.92E-03	PGR,PTN,TP53
	HOXA10			1.546	3.64E-03	AQP1,EMX2,HOXA11,HOXA5,HSP90AA1,IGFBP5,MEIS2,MFNG,PDPN, PIK3R1,PROS1
	HOXA11			2.81	3.26E-03	HOXA11,SIX2,ZNF192
	TBX3			3.031	2.26E-02	SMPX,TP53
LR7>LR3	FOS	Activated	2.588		3.34E-03	BCAR3,CD164,CFLAR,COL1A1,FLRT2,FZD5,GAS2,GSK3B,GSR,HSD17B10, IKBKAP,LGALS3BP,LTBP2,NCF2,NFIL3,PCYT2,PFN1,PIAS1,PLD1,SMAD5, IPARP,YWHAZ
	MYCN	Activated	2.410		5.60E-01	CAV1,COL1A1,PDIA4,XRCC3,ZYX
	TP53	Inhibited	−2.061		4.24E-03	AKAP12,AKT1,APC,APP,BTG2,CAV1,CFLAR,COL1A1,DDB2,EIF4G3,ENPP2,FLRT2,GSK3B,GSR,IRF7,MAPK1,MBNL2,MED13L,MUC2,MX1,NFKBIA,PLEKHB2,POLR3E,PPP1CC,PPP5C,PROSC,PSEN1,RFWD2,SH3BGRL2, SHC1,SPHK1,SREBF1,TAGLN2,TFPI2,TMEM97,VCL,ZYX
LR3>LR7	CEBPA	Inhibited	−2.445		1.90E-02	ARHGEF5,CEBPD,F9,ITGB5,LCK,MYF6,NR4A2,PLOD2,PTPRC,TBXAS1, THBD,VDR
	IKZF1			1.451	3.46E-03	CYP2J2,FGFR1,LCK,MLL,NOTCH1,NOTCH3,UACA
	NR3C1			1.603	8.63E-03	BCL2L11,BHLHE40,BMF,BRWD1,CXCL11,DNAJC15,DUT,ENC1,FN1,NR3C1,PAWR,PHF17,PPP3CC,PRPF40A,PTP4A2,RBMS2,STAT3,STAT5A,THBD, UNC13B
	EZH2			1.647	2.54E-04	ANXA6,BCL2L11,CDK6,CSNK1A1,CXCL11,EZH2,IKZF1,LYPD6B,RBM5, SATB1,TRIM38
	SREBF1			−2.24	6.91E-04	ACSL1,AK4,CYB5A,DARS,GSR,IDH1,NCF2,RDH11,SREBF1,STAR,TM7SF2, TMEM97
	PPARGC1A			−1.933	3.11E-02	DLAT,ESRRA,NDUFS1,PPARGC1A,SDHA,SREBF1,TXN2
	ESRRA			−1.373	4.65E-02	ACSL1,CYB5A,CYC1,ESRRA,PPARGC1A,SIRT1

1 z-score indications significance of prediction of activation status of the respective transcription factor; it is predicted to be activated at z-score ≥2 or inhibited at z-score ≤−2:

2 overlap p-value: indicates significance of the association between the respective transcription factor and the predicated target genes showing differential expression in the experiment; threshold p≤5.0E-02.

### Expression of epigenetic modulators and global DNA methylation in endometrium

Epigenetic changes can produce expression and functional alterations in tissues and organisms. We wished to determine whether variations existed in expression of DNA methyltransferases *DNMT1*, *DNMT3a*, and *DNMT3b* as a result of day of estrous and/or endometrial receptivity, and whether such changes affected global DNA methylation in endometrium. Therefore, we assessed transcript abundance of these three genes as epigenetic modulator enzymes and the relative global DNA methylation of individual endometrial samples of the experimental groups at days 3 and 7 of the estrous cycle. No significant differences in transcript abundance or DNA methylation level were found between receptivity groups. However, expression of *DNMT3a* was significantly different between days 3 and 7 (p = 0.008) (Table S5). Additionally, expression of both *DNMT1* and *DNMT3b* differed between days 3 and 7 at p = 0.080 and p = 0.063, respectively (Table S5). Further, the interaction of receptivity and day of cycle affected the transcript abundance of *DNMT1* at p = 0.013, of *DNMT3a* at p = 0.087, and of *DNMT3b* at p = 0.051, as well as the % DNA methylation at p = 0.024 (Figures S1 and S2). Accordingly, least-squares means revealed a trend of decreasing global DNA methylation from days 3 to 7 of the estrous cycle in HR, but increasing methylation in LR group. The overall correlation of *DNMT1*, *DNMT3a*, and *DNMT3b* transcript abundances and the relative global DNA methylation was not significant. However, the expression level of *DNMT1* at day 7 of estrous significantly correlated with the relative global DNA methylation (R^2^ = 0.554; p = 0.021, N = 18).

## Discussion

Embryo implantation is a complex process. Successful implantation requires appropriate communication between the embryo and the maternal endometrium, a tissue that is crucial for the establishment and maintenance of pregnancy [2], [9]–[11]. A receptive endometrium and a normal and functional embryo at the blastocyst developmental stage are prerequisites of a successful implantation, which is characterized by a synchronized dialogue between maternal and embryonic tissues [12]. However, the regulation of implantation remains incompletely understood. On one hand, the process of implantation is driven by embryonic signals conveyed through various proteins, cytokines, and growth factors that systematically modulate maternal anatomy, endocrinology, immunology, and physiology to create an environment conductive to fetal development and survival [13], [14]. On the other hand, however, inadequate uterine receptivity is thought to be responsible for two-thirds of implantation failure [15]. A recent report described transcriptional changes in the endometrium during the menstrual cycle and embryonic implantation in human [16]. Similarly, expression profiling has been performed during both the estrous cycle [6] and implantation in cattle [17], even accounting for different biotechniques used to derive embryos [18]. These reports identified the endometrium as an early sensor for the presence and implantation of embryos in the uterus [8], [18]. The microarray data reported here indicate that endometrial expression patterns and alterations of transcript abundance are indicative of the receptivity of the endometrium and, thus, of pregnancy success.

### Comparisons of high receptive and low receptive endometria

Our microarray analysis revealed that many biological processes and molecular functions differed between receptivity groups at day 3 or day 7 of estrous. Indeed, more differences were found in gene expression profiles at day 3 of estrous than at day 7. The data suggest that the influence of hormones (estrogen and progesterone) through the estrous cycle plays a significant role in implantation success.

Several molecules involved in integrin signaling, cytoskeleton signalling, and Rac signaling were enriched in the high receptive endometrium at the beginning of the estrous cycle. Transcripts of these pathways were found to increase during the window of implantation and were proposed as markers for the implantation process [19]–[23]. Integrins participate in cell–matrix and cell–cell adhesion in many physiologically important processes. Cellular aggregation leads to the recruitment of a network of cytoskeletal proteins and intracellular signaling complexes. The acquisition of adhesion ligands, together with the loss of inhibitory components that may act as a barrier to an attaching embryo, contribute to endometrial receptivity [22], [23]. In cattle a number of mRNAs coding for components of the cytoskeleton and extracellular matrix proteins were as enriched at estrous [6]. Our study shows that genes of molecular signaling pathways that determine the communication skills of the endometrium are differentially expressed between the high and low receptive groups at a time early in advance of implantation.

Many transcripts showed lower abundance in the low receptive endometrium at day 3 of estrous belong to biofunctions relevant to energy supply, including mitochondrial pathways. This indicates that defects in these pathways may contribute to the development of low receptive endometrium. The role of estrogen in directly and indirectly affecting mitochondrial activity is well established and was functionally exploited in experiments using estrogen receptor-α knockout mice and ovariectomized rats in combination with estrogen supplementation [24], [25]. Others reported that estrogen increases NRF-1 transcription, resulting in a coordinated increase of expression of nuclear- and mitochondrial-encoded genes and mitochondrial respiratory activity in MCF-7 breast and H1793 lung adenocarcinoma cells [26]. Recently, gene-set enrichment pathway analyses identified mitochondrial dysfunction as a biological process related to timing of menopause in human, coinciding with a reduction in estrogen levels [27]. Ovarian hormones significantly affect regulation of mitochondrial biogenesis and activity and, in turn, may affect the receptivity of the endometrium and finally pregnant. Indeed, the observed expression differences related to mitochondrial function were prominent at day 3 of estrous, when estrogen is still the dominant hormone.

Here, we also observed differential expression of many transcripts among high and low receptive endometrial samples on day 7 of estrous, in line with our previous study [8]. In addition, IPA particularly indicated many inflammatory and immune mediators playing a role in T- and B cell signaling and NF-κB signaling that had more abundant transcripts at this time point in the high receptive endometrium. The endometrium is a dynamic tissue that undergoes cycles of proliferation, differentiation, breakdown, and repair [28], [29]. Further, in human the implantation window is characterized as an inflammatory event associated with increased expression of inflammatory mediators and immune cell infiltration [29]. On the endometrial side, preparation for implantation is necessary to receive a blastocyst through mediation by immune cells, cytokines, growth factors, chemokines, and adhesion molecules [29]–[31]. The physiological events of implantation and menstruation display features of inflammation, tightly regulated by estrogen and progesterone [32]. At day 7 of the estrous cycle in cattle, progesterone is increased and begins to regulate decidualization of the endometrium, the number of uterine natural killer cells, and endometrial chemokine and cytokine expression. The fact that NF-κB activity is regulated in the endometrium throughout the sexual cycle as well as a developing pregnancy indicate a functional link between estrogen and progesterone levels, NF-κB signaling, and endometrial receptivity [33].

Samples of low receptive endometrium at day 7 of the estrous cycle revealed an increase in expression of transcripts assigned to biological functions of inhibition of angiogenesis and NRF2-mediated oxidative stress. Angiogenesis is a key feature of endometrial development. Accordingly, associated factors and signals, like VEFG and its receptors, play important roles in implantation and maintenance of pregnancy alike [34]. Here, expression of *GUCY1A3* and *GUCY1B3* was lower in the low receptive endometrium at day 7. GUCY1A3 and GUCY1B3 affect angiogenesis by markedly reducing the content of cGMP, an upstream mediator of VEGF expression in glioma cells, and the expression of VEGF [35]. Differential expression of genes involved in angiogenesis among the high and low receptive groups reflect a poor preparation of the endometrium to receive and supply an embryo. Similarly, for transcripts involved in oxidative stress, several lines of evidence indicate that oxidative stress is associated with pregnancy loss or complications of various pathological backgrounds [36]–[38]. Correspondingly, we found a higher expression of oxidative stress response genes in the low receptive group at day 7 of estrous.

Taken together, the analysis of transcriptional differences among the high and low receptive groups at either day 3 or day 7 of the estrous cycle points to deficiencies of molecular cell signaling pathways and energy supplying mitochondrial pathways at very early, estrogen-dominated stages of the estrous cycle—in advance of potential implantation of an embryo—in the low receptivity group. At day 7 of the estrous cycle, at decreasing estrogen levels and increasing progesterone levels, the results of the present study reveal a biological function of inflammatory signaling for receptivity of the endometrium. Moreover, transcripts associated with response to oxidative stress as well as inhibition of angiogenesis are more abundant in samples from the low receptivity group, indicating that mitochondrial metabolic homoeostasis of the endometrium and tissue expansion towards a better preparation for implantation and placental function contribute to improved receptivity and pregnancy success.

### Temporal regulation from day 3 to day 7 of estrous cycle

Throughout the estrous cycle the bovine endometrium exhibits characteristic morphological and functional changes that are mainly induced by estrogen and progesterone and coincidence with expression changes in the endometrium [6]. Here, we considered only those transcripts showing varied alteration of expression from day 3 to day 7 of estrous between high receptive and low receptive endometrium, and not those transcripts that were consistently regulated in both experimental groups due to physiologically developmental processes in the estrous cycle.

We observed a number of biofunctions showing divergent longitudinal regulation in the high and low receptive groups that corresponded to the observations made between the groups at either day 3 or day 7 of the estrous cycle. In the high receptivity group transcriptional changes from day 3 to day 7 were dominated by processes related to cell proliferation and growth, which were lacking in the low receptivity group and accumulate in more mature, expanded, highly receptive tissues, for example, showing higher inositol phosphate metabolism as a indicator of cell growth. Moreover, temporal regulation of transcripts related to mitochondrial dysfunction in the LR group is insufficient to compensate deficiencies evident at day 3 in the low receptivity group, as indicated by continued higher expression of genes responding to oxidative stress at day 7 in the LR group. Similarly, the divergent temporal regulation of transcripts of genes related to immune, inflammatory, and stress-relevant pathways potentially reflects compensatory effects. However, more immune and stress genes were dominant at day 7 compared to day 3 of estrous in high receptive endometrium. In the LR group higher expression was found for genes belonging to prolactin signaling pathways and shared with immune or stress signaling pathways at day 3 of estrous.

The results indicate that longitudinal changes reflect differences in the relative abundance of numerous transcripts of pathways related to cell communication, mitochondrial function, tissue proliferation and angiogenesis, and inflammatory and immune response at the two distinct time points. The divergent temporal regulation points to biofunctions and pathways that are inhibited in the LR group. Those pathways were identified where relative compensation and regulation towards balancing among both groups is evident. The endometrial tissue of the HR group at day 7 seems better prepared for implantation due to the recruitment of genes relevant for proliferation and growth, energy supply, and inflammatory and immune responsiveness. The stimulation of theses biofunctions partly depends on effects of estrogen and progesterone that undergo major changes in concentration early in the estrous cycle.

### Transcription factor analysis

Tumor protein p53 (TP53) is the most prominent transcription factor that we linked to the regulation of many genes in this study in a direction consistent with activation; TP53 was also found to be regulated itself. TP53 regulates cell division and has well-established functions in controlling cell cycle arrest and apoptosis [39]. Recently, p53 has been shown to be involved in reproduction by regulating the expression of the leukemia inhibitory factor gene in the maternal endometrium [40], [41]. A single nucleotide polymorphism (SNP) in the human p53 gene is associated with reduced fertility [40]–[42]. Additional, p53 in human induces expression of the pregnancy-supporting human chorionic gonadotropin (hCG) *CGB7* [43]. In the present study TP53 was predicted as an activator for genes that showed higher transcript abundance in the high receptive endometrium between day 3 and day 7 as well as between HR and LR at day 3 of estrous. Quantitative real-time PCR of individual endometrium samples confirmed highly significant differences (p<0.0001) between the high receptive and low receptive groups. TP53 was predicted as a inhibitor of the genes that more frequent transcripts at day 7 of the estrous cycle in the low receptive group. With these results, we propose a new link between TP53 transcriptional activity and cattle reproduction.

FOS is an early transcription factor that has been reported to be related to estradiol-dependent cell proliferation and may play a role in the molecular mechanisms of estrogen action on the induction, promotion, or progression of endometriosis [44], [45]. In the present study FOS was predicted as an inhibitor for downstream genes in the high receptive group at day 7 of estrous and was predicted as activator for downstream genes in the low receptive group. These data suggested that the expression pattern of downstream target genes of FOS may be a biological marker for pregnant success.

Some transcription factors were themselves differentially expressed, such as *HOXA5, HOXA10,* and *HOXA11*, which were more highly expressed at day 3 of estrous in the high receptive group. *HOX* genes are transcriptional regulators that play an essential role in determining tissue identity during embryonic development. In particular, the role of transcription factors *HOXA10* and *HOXA11* and their downstream genes in endometriosis and related infertility has been shown [46]–[48]. *HOXA10* function is essential for regulating endometrial development during the menstrual cycle and in establishing conditions necessary for implantation in the human. It regulates a variety of downstream genes, including cell adhesion molecules, signal transduction factors, and metabolic mediators, and affects both endometrial stromal cell (ESC) proliferation and epithelial cell morphogenesis [47], [49], [50].

Another transcription factor that was temporally regulated in the LR group was *EZH2*. *EZH2* is a member of the polycomb group of genes and is important in cell cycle regulation. Functions associated with increased expression of *EZH2,* like invasive growth in prostate [51] and breast cancer [52] as well as endometrial carcinoma [53] point to its role in endometrium proliferation early in the estrous cycle.

For some of the transcription factors and targets molecules abundant evidence for potential roles in endometrial function comes from previous studies in humans and mice. Their corresponding role in cattle endometrium as a molecular marker of receptivity was demonstrated here. Moreover, we listed transcription factors that may play significant roles in implantation success that were identified for the first time here in the cattle endometrium.

### Expression of epigenetic modulators and global DNA methylation in endometrium

Aberrant expression of endometrial genes or proteins due to inappropriate epigenetic modification may causally contribute to the failure of embryo implantation [54]. Significant changes in the expression of DNA methyltransferases (DNMTs) have been observed in human endometrium during the menstrual cycle [55], and in endometrial pathologies [56], [57]. DNMTs have regulatory functions in gene expression by mediating the methylation of cytosine, i.e., by affecting the epigenetic regulation of gene expression. These findings suggest a role for DNMTs in the growth and differentiation of the endometrium, including effects on the receptivity of the uterus. Epigenetic events controlling the transcriptional regulation of genes involved in endometrial function during the estrous cycle and early pregnancy remain less clear, with the reports of low levels of global DNA methylation in endometrium, likely controlled by the observed moderate *DNMT3b* expression [58]. In the present study, we did not find significant differences in global DNA methylation in the bovine endometrium during estrous or among receptivity groups. This is in accordance with a previous study [58]. However, we found significantly different expression levels of *DNMT1* and global DNA methylation depending on the interaction between day within estrous cycle and receptivity group. This finding indicates that the exact timing of epigenetic modification during the estrous cycle is associated with receptivity and pregnancy success. Thus, epigenetic regulation of the bovine endometrium plays a critical role in pregnancy success but is complex and deserves further investigation.

### Conclusion

We provide a comprehensive inventory of genes and functional networks expressed in cattle endometrium and representing the maternal part of the embryo-maternal cross-talk at early stages of the estrous cycle. These genes and networks are associated with uterine receptivity and may be predictive of the success of pregnancy in bovine. It was previously shown that differential expression among high and low receptive endometrial tissues at day 7 of estrous is much more pronounced than at day 14, when progesterone reaches peak levels [8]. Here we show that, even very early in the estrous cycle at day 3, when estrogen is still the dominant ovarian hormone, considerable differences in the abundance of transcripts between HR and LR are obvious, particularly concerning genes involved in cell signaling and mitochondrial pathways. At day 7, at decreasing estrogen and increasing progesterone levels, transcripts related to inflammatory signaling, oxidative stress, and vascularization are differentially expressed. The study indicates that cell communication skills, mitochondrial metabolic homoeostasis, and tissue expansion towards a better preparation for implantation and placental functions contribute to improved receptivity and pregnancy success. Ovarian hormones are known to play a significant role in regulation of endometrium development via various biological pathways. Key components of these molecular pathways are known to be dependent on ovarian hormones promoting the role of hormone levels and tissue responsiveness to the hormones for uterine receptivity. Many of the transcription factors that play a significant role in pregnant success in various species were confirmed or identified for the first time here in the bovine endometrium. Additionally, epigenetic changes appear to play a key role in developmental processes that occur during estrous and the establishment of pregnancy. In our study, the interaction of time point and receptivity was significant for global DNA methylation, leading to divergent trends of decreasing or increasing DNA methylation in the HR or LR group, respectively. This indicates that the temporal epigenetic regulation of bovine endometrium plays a critical role in receptivity.

## Materials and Methods

### Animal handling and tissue collection

Animal care and tissue collection were performed according to guidelines of the German Law of Animal Protection and with approval by the institutional Animal Care Committees of the Leibniz Institute for Farm Animal Biology and the University of Bonn. After estrous synchronization of cyclic Simmental heifers and ascertainment of estrous via examination of common visible signs, endometrial biopsies were collected using a cytobrush technique as described [8]. In brief, a brush (length: 20 mm, diameter: 6 mm, from Gynobrush, Heinz Herenz, Hamburg, Germany) encased in a one-way catheter was inserted into the uterus, passing vagina and cervix, and stroked along the uterine wall. Brush and adhering tissue were retracted into the catheter and pulled out of the animal. Endometrial tissues attached to brushes were immersed in RNAlater RNA Stabilization Reagent (Sigma-Aldrich, Taufkirchen, Germany) and stored at −80°C. This procedure was performed on days 3 and 7 of the estrous cycle during the pre-transfer period.

### Pregnancy diagnosis

At day 7 of the estrous cycle following the cycle of biopsy *in vitro*-produced blastocysts all scored as grade I according to the Manual of the International Embryo Transfer Society (IETS) were transferred to the estrous synchronized Simmental heifers [8]. After transfer of the blastocysts the animals were monitored for their pregnancy status via the measurement of blood progesterone levels (up to day 24) and ultrasonography examinations on day 28. Tissue samples collected on days 3 and 7 of the pre-transfer cycle were then retrospectively grouped according to pregnancy success. Those animals with a detectable embryo on day 28, i.e., being pregnant, were assigned to the high receptivity group (HR); cows in which no embryo could be detected on day 28 were assigned to the low receptivity group (LR).

### RNA isolation and quality control

Samples frozen in RNAlater were thawed at room temperature so brushes could be removed. Endometrial tissue, which stuck firmly to the bristles of the brushes, were carefully scraped off using sterile needles. Total RNA of the biopsies was isolated by Tri-Reagent-phenol-chloroform extraction (Sigma-Aldrich, Taufkirchen, Germany) according to manufacturer's protocol. To remove any DNA, a DNase (Qiagen, Hilden, Germany) treatment and purification using the RNeasy Mini Kit (Qiagen, Hilden, Germany) were performed. RNA was solubilized in 15 µl RNase-free water and stored at −80°C to be used for both the subsequent microarray analysis and qRT-PCR. To assess RNA integrity, 1 µl of RNA was loaded onto 1% agarose gel containing ethidium bromide. To rule out DNA contamination, PCR was performed on all RNA samples using primers for the bovine glyceraldehyde-3-phosphate dehydrogenase (GAPDH) gene. RNA concentration and quality were assessed using a Nanodrop 1000 Spectrophotometer (ThermoFisher Scientific, Schwerte, Germany).

### Whole-genome expression profiling

Four groups of endometrial biopsies were compared; these were obtained on days 3 and 7 of the pre-transfer cycle but retrospectively defined as high or low receptive endometria based on pregnancy success in the transfer cycle. The groups were as follows: high-receptive for day 3 and day 7, HR3 and HR7, and low-receptive for day 3 and day 7, LR3 and LR7. Gene expression profiling was conducted on 15 samples per group, which were assigned to 3 pools of 5 samples per group. In brief, total RNA was used for target preparation for microarray hybridization. Using Affymetrix protocols, 500 ng of total RNA were reverse-transcribed into cDNA, transcribed into cRNA, and labeled using Affymetrix One cycle synthesis and labeling kit (Affymetrix, Santa Clara, CA, USA) to prepare antisense biotinylated RNA targets. Endometrium expression patterns were produced using 12 GeneChip Bovine Genome Arrays (Affymetrix, Santa Clara, CA, USA). Quality of hybridization was assessed in all samples following manufacturer's recommendations. Data were analyzed with the Affymetrix GCOS 1.1.1 software using global scaling to a target signal of 500. Data were processed with MAS5.0 to generate cell intensity files (present or absent). Quantitative expression levels of the present transcripts were estimated using the PLIER algorithm (Probe Logarithmic Intensity Error) for normalization that was implemented in the Expression Console software (Affymetrix, Santa Clara, CA, USA). All probe sets with a standard deviation <0.2 were filtered out, and remaining data were used for analysis of variance, using pregnancy status and day of estrous cycle as fixed effects in SAS base program JMP Genomics. Essentially the SAS MIXED procedure fits linear models on a row-by-row basis to pre-normalized data. The adjusting for multiple tests across the Type 3 tests for all of the fixed effects was calculated using the post hoc Tukey-Kramer test. For controlling false discovery rate (which is less restrictive than false positive rate), we choose the FDR according to Benjamini and Hochberg, 1995, which is subsequently given as ‘adjusted P values’. Microarray data were deposited in the Gene Expression Omnibus public repository [GEO accession number: GSE36079; GSM880824–GSM880835].

### Pathway analysis and transcription factor analysis

Annotations of the Affymetrix identifiers to human gene symbols are based on Hintermair [59] supplemented with additional information obtained from the NetAffx annotation provided by Affymetrix. The list of significant transcripts based on the effect was analyzed, referring to predefined pathways and functional categories of the Ingenuity Knowledge Base using Ingenuity Pathway Analysis (IPA). Canonical pathways were identified from the Ingenuity Pathway Analysis library. The significance of the association between the dataset and predefined pathways and functional categories was measured by Fisher's exact test. The goal of the IPA transcription factor analysis was to identify the cascade of upstream transcriptional regulators that can explain gene expression changes in endometrial samples. The transcriptional regulator analysis was based on prior knowledge of expected effects between transcriptional regulators and their target genes stored in the Ingenuity Knowledge Base. Details of the transcriptional regulator analysis were found in manufacturer's guide (www.ingenuity.com; release December, 2011). In brief, for each potential transcriptional regulator, two statistics were computed. Overlapping p-values (Fisher's exact test p-value) indicate the significance of genes in the dataset that are downstream of the transcription factor. Z-score was used to identify transcriptional regulators that are able to explain gene expression changes and infer their activation state. The bases for this prediction are edges (relationships) in endometrial samples that are associated with a literature-derived regulation direction that can be either ‘activating’ or ‘inhibiting’.

### Reverse transcription and quantitative real-time PCR

cDNA was synthesized from 2 µg of total RNA with random primers (Promega), oligo d(T) 13VN (Sigma-Aldrich, Taufkirchen, Germany), and the SuperScriptII Reverse Transcriptase Kit (Life Technologies GmbH, Darmstadt, Germany) following the manufacturer's guide. Quantitative real-time PCR was performed using the LightCycler 480 Real-Time PCR System (Roche, Genzach, Germany). Amplification was conducted in duplicate according to supplier's instructions. Reactions were performed in a final volume of 10 µl using 5.0 µl of LightCycler 480 SYBR Green I Master (Roche, Grenzach, Germany), 2.0 µl of distilled water, 10 µM of each primer, and 40 ng cDNA. Temperature profiles comprised an initial denaturation step at 95°C for 10 min, and 40 cycles with denaturation at 95°C for 15 sec, annealing at 60°C for 10 sec, and extension at 72°C for 15 sec. For all assays threshold cycles were converted to copy numbers using a standard curve generated by amplifying serial dilutions of an external PCR standard (10^7^ - 10^2^ copies). After completion of amplification protocol all samples were subjected to melting curve analyses and gel electrophoresis. Primers were obtained from Sigma-Aldrich. Primer sequences are listed in Table S6. Expression levels were normalized to *GAPDH*, *RPS13*, and *SUZ12*. *SUZ12* is accepted as the most appropriate control gene for use in bovine endometrium during early pregnancy or the estrous cycle [60]. *RPS13* and the well-known house keeping gene GAPDH showed no regulation for day of estrous cycle or pregnancy groups in our array data [8]. Eleven target genes (*DDX46*, *KRT5*, *PTGES*, *PTGS2*, *HPGD*, *ESRRA*, *TP53*, *PIP*, *AP2S1*, *SLK*, *ATP1B3*) were chosen for validation of microarray results by qRT-PCR. These genes either belong to molecular routes previously known (hormone signaling: *PTGES*, *PTGS2*, *PIP*, *ESRRA*, *HPGD*) or indicated here to be relevant for endometrium receptivity. Nine endometrial samples per group were used.

### Global DNA Methylation

To investigate global DNA methylation in endometrium at days 3 and 7 of estrous, we used the Imprint Methylated DNA Quantification Kit (Sigma-Aldrich, Taufkirchen, Germany) following the supplier's protocol. A total amount of 100 ng DNA was applied. In this assay, 5-methylcytosine-modified genomic DNA is recognized by 5-methylcytosine antibody, and the bound DNA is quantified in a colorimetric reaction. Positive (methylated) and negative (unmethylated) control DNA were supplied with the kit. Relative global DNA methylation level was calculated as percentage relative to methylated control DNA. DNA from 9 individual endometrium samples per group was used. Individual expression levels of *DNMT1*, *DNMT3a*, and *DNMT3b*, coding for DNA methyltransferases that are responsible for methylation, were quantified with real-time PCR.

### Statistical analyses of quantitative real-time PCR and global DNA Methylation

Statistical analyses of normalized real time PCR data and Global methylation data were performed by ANOVA with the SAS 9.2 software package for windows (SAS Institute, Inc.). The procedure GLM using the method of least squares to fit general linear models and a post hoc Tukey-Kramer test were applied. The fixed effects of receptivity group, day of oestrus and their interaction were considered in the model. Statistical dependence between microarrays data and real-time PCR data was estimated using Spearman's rank correlation coefficient. All data are given as least-squares means (LSMEANS). A p-value of <0.05 was considered to be statistically significant.

## Supporting Information

Figure S1 **Relative amounts of DNMT1, DNMT3a, and DNMT3b transcripts in the HR and LR group at day 3 and day 7 of estrous cycle.**
(TIF)

Figure S2 **Global DNA methylation in the HR and LR group at day 3 and day 7 of estrous cycle.**
(TIF)

Table S1 **List of transcripts with different abundance in the HR and LR groups at day 3 of the estrous cycle.**
(XLS)

Table S2 **List of transcripts with different abundance in the HR and LR groups at day 7 of the estrous cycle.**
(XLS)

Table S3 **List of transcripts with different changes of abundance between day 3 and day 7 of the estrous cycle in the HR groups.**
(XLS)

Table S4 **List of transcripts with different changes of abundance between day 3 and day 7 of the estrous cycle in the LR groups.**
(XLS)

Table S5 **Significance of differences (p-value) of transcript amounts of** ***DNMT1*** **,** ***DNMT3a*** **, and global DNA methylation in endometrium depending on receptivity group or days of estrous cycle and the interaction.**
(DOC)

Table S6 **qRT-PCR primers of candidate genes and housekeeping genes.**
(DOC)
