# Supplementary figures and images for: Gene Expression and DNA-Methylation of Bovine Pretransfer Endometrium Depending on Its Receptivity after In Vitro-Produced Embryo Transfer
Source: PLoS One. 2012 Aug 27;7(8):e42402. doi: 10.1371/journal.pone.0042402 (PMC3428322; doi:10.1371/journal.pone.0042402)

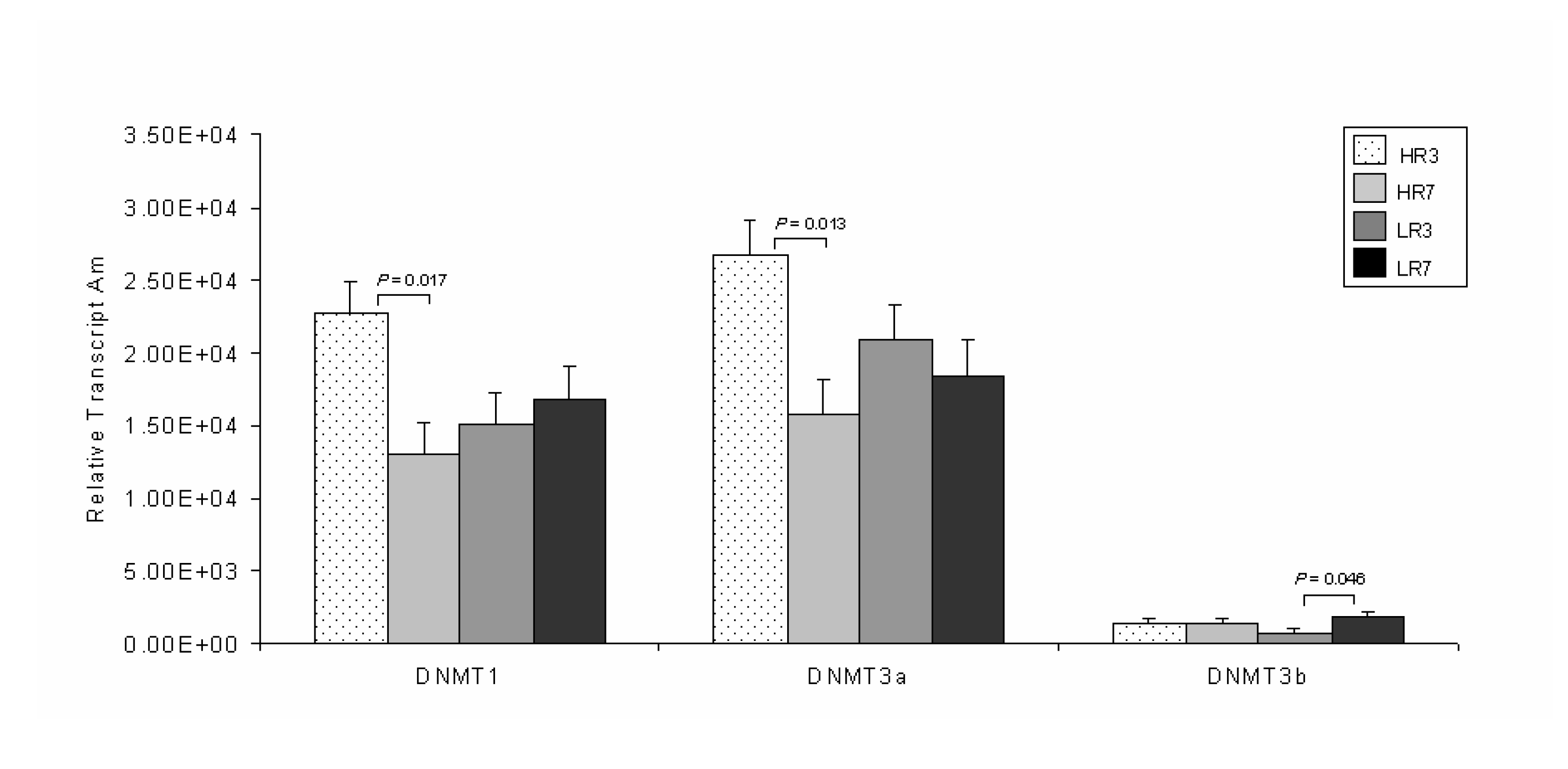

Supplement: Figure S1 — Relative amounts of DNMT1, DNMT3a, and DNMT3b transcripts in the HR and LR group at day 3 and day 7 of estrous cycle. (TIF) [file pone.0042402.s001.tif]

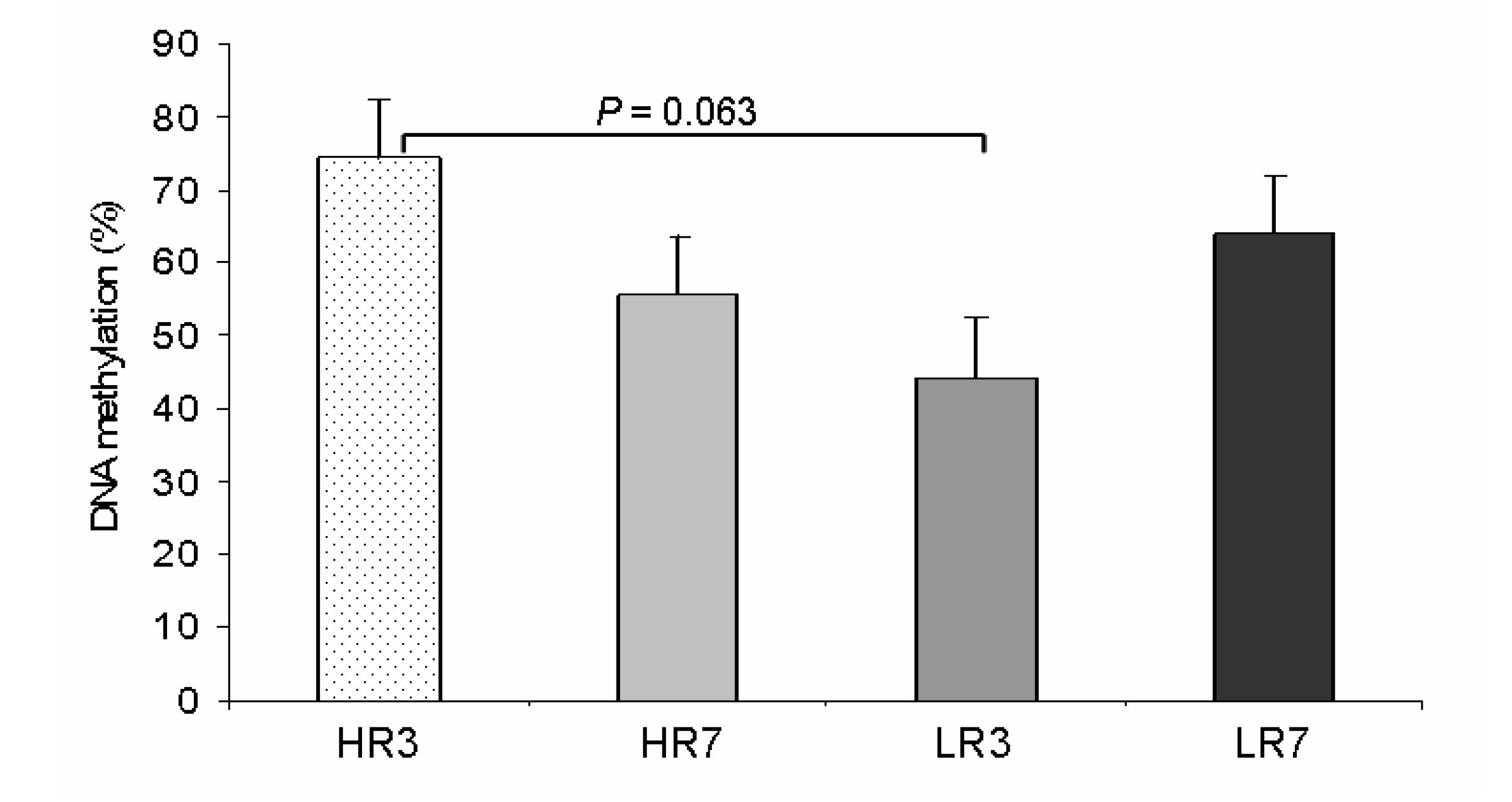

Supplement: Figure S2 — Global DNA methylation in the HR and LR group at day 3 and day 7 of estrous cycle. (TIF) [file pone.0042402.s002.tif]
