# Supplementary material for: Gene Expression and DNA-Methylation of Bovine Pretransfer Endometrium Depending on Its Receptivity after In Vitro-Produced Embryo Transfer
Source: PLoS One. 2012 Aug 27;7(8):e42402. doi: 10.1371/journal.pone.0042402 (PMC3428322; doi:10.1371/journal.pone.0042402)
Supplement: Table S5 — Significance of differences (p-value) of transcript amounts of DNMT1 , DNMT3a , and global DNA methylation in endometrium depending on receptivity group or days of estrous cycle and the interaction. (DOC) [file pone.0042402.s007.doc]

**Supplementary Table S5.** Significance of differences (p-value) of transcript amounts of *DNMT1*, *DNMT3a*, and global DNA methylation in endometrium depending on receptivity group or days of estrous cycle and the interaction.

|  | LSMEAN of relative expression levels in receptive group (R) | |  | LSMEAN of relative expression levels in estrous cycle (D) | |  | Interaction  of R*D |
| --- | --- | --- | --- | --- | --- | --- | --- |
|  | HR | LR | p-value | d3 | d7 | p-value | p-value |
| DNMT1 | 17908.5 | 15977.5 | 0.385 | 18926.7 | 14958.4 | 0.080 | 0.013 |
| DNMT3a | 21264.4 | 19656.5 | 0.505 | 23811.6 | 17109.4 | 0.008 | 0.087 |
| DNMT3b | 1407.6 | 1375.6 | 0.918 | 1095.4 | 1687.9 | 0.063 | 0.051 |
| % methylation | 65.1 | 54.0 | 0.179 | 59.2 | 59.9 | 0.927 | 0.024 |
