# Supplementary material for: Gene Expression and DNA-Methylation of Bovine Pretransfer Endometrium Depending on Its Receptivity after In Vitro-Produced Embryo Transfer
Source: PLoS One. 2012 Aug 27;7(8):e42402. doi: 10.1371/journal.pone.0042402 (PMC3428322; doi:10.1371/journal.pone.0042402)
Supplement: Table S6 — qRT-PCR primers of candidate genes and housekeeping genes. (DOC) [file pone.0042402.s008.doc]

**Supplementary Table S6.** qRT-PCR primers of candidate genes and housekeeping genes.

| **gene** | **primer forward 5'-3'** | **primer reverse 5'-3'** | **amplicon** |
| --- | --- | --- | --- |
| KRT5 | ATCAACAACCTCAGGAGACAG | GCATCCACATCCTTCTTCAG | 175 bp |
| PTGES | GACGCTCAGAGACATGGA | GAAAGAGTAGACAAAGCCCAG | 128 bp |
| PTGS2 | GTGTGAAAGGGAGGAAAGAG | GCAAACATCAGATTTGTGCC | 105 bp |
| DDX46 | AAGCAGGGATAGAGACAGGA | TCTTCTTCGTTCTCGACTTCTA | 129 bp |
| HPGD | AAAGTACCTCATTCTGCCTG | CAGTTCCTCATATTCAATCCAC | 179 bp |
| ESRRA | CCTGGTCCTGGATGAAGAG | GAGTCTGAATTGGCGAGG | 149 bp |
| AP2S1 | ACGACAACAACCTGGCCTAC | GCCAGGAACATCTCATCCAC | 139 bp |
| DNMT3a | AATTGCTACAGGGCTTCTGG | CCCATTCCTGGATATGCTTC | 170 bp |
| DNMT3b | AAGGACTGGAGTGTGCGTCT | TTTGATGCCCAGTTCTTTGA | 174 bp |
| DNMT1 | TCCATGGTCCTGAAGCTGAC | GGTGCGAACACATGCAAC | 179 bp |
| GAPDH | ACCCAGAAGACTGTGGATGG | CAACAGACACGTTGGGAGTG | 178 BP |
| SUZ12 | GAACACCTATCACACACATTCTTGT | TAGAGGCGGTTGTGTCCACT | 130 bp |
| RPS13 | CTACAAACTGGCCAAGAAAGG | TCAGGGAGATCAGGAGCAAG | 147 bp |
| SLK | TGGGCAGAAGTTAAGGCTACC | TGAAAACTTTGAATGCTGAACC | 138 bp |
| PIP | GGTTTGCAAATCCCAGAAGA | AGATCTGAGGGAATGCATGG | 133 bp |
| ATP1B3 | CTTGAAGCATGCAGTGGTGT | AAATGCTGTGCTTTCGCTCT | 150 bp |
| TP53 | GGTTCACCAAGGGGTTATGA | CTTTTGTGGGTGCTGGTTCT | 134 bp |
